# Mutation Analysis of *NR5A1* Encoding Steroidogenic Factor 1 in 77 Patients with 46, XY Disorders of Sex Development (DSD) Including Hypospadias

**DOI:** 10.1371/journal.pone.0024117

**Published:** 2011-10-20

**Authors:** Slimane Allali, Jean-Baptiste Muller, Raja Brauner, Diana Lourenço, Radia Boudjenah, Vasiliki Karageorgou, Christine Trivin, Henri Lottmann, Stephen Lortat-Jacob, Claire Nihoul-Fékété, Olivier De Dreuzy, Ken McElreavey, Anu Bashamboo

**Affiliations:** 1 Université Paris Descartes, Faculté de médecine and AP-HP, Hôpital Bicêtre, Unité d'Endocrinologie, Pédiatrique, Le Kremlin Bicêtre, France; 2 Human Developmental Genetics, Institut Pasteur, Paris, France; 3 AP-HP, Hôpital Necker-Enfants Malades, Service d'explorations fonctionnelles, Paris, France; 4 AP-HP, Hôpital Necker-Enfants Malades, Service de chirurgie viscérale pédiatrique, Paris, France; 5 Université Paris Descartes, Paris, France; 6 AP-HP, Hôpital Bicêtre, Service de Chirurgie Pédiatrique, Le Kremlin Bicêtre, France; University of Muenster, Germany

## Abstract

**Background:**

Mutations of the *NR5A1* gene encoding steroidogenic factor-1 have been reported in association with a wide spectrum of 46,XY DSD (Disorder of Sex Development) phenotypes including severe forms of hypospadias.

**Methodology/Principal Findings:**

We evaluated the frequency of *NR5A1* gene mutations in a large series of patients presenting with 46,XY DSD and hypospadias. Based on their clinical presentation 77 patients were classified either as complete or partial gonadal dysgenesis (uterus seen at genitography and/or surgery, n = 11), ambiguous external genitalia without uterus (n = 33) or hypospadias (n = 33). We identified heterozygous *NR5A1* mutations in 4 cases of ambiguous external genitalia without uterus (12.1%; p.Trp279Arg, pArg39Pro, c.390delG, c140_141insCACG) and a *de novo* missense mutation in one case with distal hypospadias (3%; p.Arg313Cys). Mutant proteins showed reduced transactivation activity and mutants p.Arg39Pro and p.Arg313Cys did not synergize with the GATA4 cofactor to stimulate reporter gene activity, although they retained their ability to physically interact with the GATA4 protein.

**Conclusions/Significance:**

Mutations in *NR5A1* were observed in 5/77 (6.5%) cases of 46,XY DSD including hypospadias. Excluding the cases of 46,XY gonadal dysgenesis the incidence of *NR5A1* mutations was 5/66 (7.6%). An individual with isolated distal hypopadias carried a *de novo* heterozygous missense mutation, thus extending the range of phenotypes associated with *NR5A1* mutations and suggesting that this group of patients should be screened for *NR5A1* mutations.

## Introduction

The term Disorders of Sex Development (DSD) has been defined as ‘congenital conditions in which the development of chromosomal, gonadal, or anatomical sex is atypical.’ Therefore DSD constitutes a spectrum of disorders that affect the genito-urinary tract and the endocrine-reproductive system [1]. 46,XY DSD includes the conditions of 46,XY partial or complete gonadal dysgenesis, and undervirilisation or undermasculinization of an XY male. Some of these phenotypes, such as 46,XY gonadal dysgenesis are relatively rare, whereas the 46,XY DSD related phenotype hypospadias is a relatively common congenital anomaly with an estimated incidence of 1∶200–1∶300 live births [2].

The etiology of hypospadias is unknown in most cases. Only approximately 5% of all cases of hypospadias are explained by rare genetic anomalies of androgen biosynthesis or androgen receptor [2]. Mutations in genes expressed in the developing male gonad including *MAMLDL1* or mutations in genes that are associated with syndromic forms of hypospadias such as *HOXA13* are rare [3], [4]. A few genetic associations have been reported, but these involved small cohort sizes and most of these studies have not been replicated [5], [6]. Although environmental factors may contribute to the formation of hypospadias *in utero* and may be linked with the proposed increase in the incidence of hypospadias in some Western countries [7], [8], the familial aggregation that is often associated with hypospadias suggests a genetic component [9].

The gene *NR5A1*, which encodes steroidogenic factor-1, is a pivotal transcriptional regulator of genes involved in the hypothalamic-pituitary-steroidogenic axis [10], [11]. During early male development *NR5A1* positively regulates the expression of two key genes involved in male sex determination and differentiation, *SOX9* (Sry-box 9), and Anti-Müllerian Hormone (AMH) [12], [13]. NR5A1 also modulates the expression of many factors involved in cholesterol mobilization and steroid hormone biosynthesis including HMG-CoA synthase, steroidogenic acute regulatory protein (StAR), 3β-hydroxysteroid dehydrogenase (3βHSD), and several cytochrome P450 steroid hydroxylase (CYP) enzymes [14]. The NR5A1 protein consists of a DNA-binding domain (DBD) of two zinc fingers, a hinge region, a ligand-binding domain (LBD), and two activation function domains [15]. NR5A1 is expressed in the Sertoli and Leydig cells of the developing testis and in Sertoli cells of the prepubertal and adult testis [16], [17]. Mice lacking *Nr5a1* show both gonadal and adrenal agenesis [11]. Human *NR5A1* mutations were first reported in association with 46,XY DSD and adrenal insufficiency and in a 46,XX girl with adrenal insufficiency [14], [18]. More recently, the range of phenotypes that are associated with *NR5A1* mutations has broadened and now includes 46,XY complete and partial gonadal dysgenesis, penoscrotal hypospadias, microphallus with anorchidia and 46,XX primary ovarian insufficiency (POI) [15], [19]–[21]. In all of these more recent cases, there is no evidence of adrenal insufficiency. In a large study on hypospadias, it was concluded that heterozygous loss-of-function mutations in *NR5A1* could be found in severe forms of hypospadias, but not found frequently in association with minor forms of hypospadias [19].

In this study, we evaluated the frequency of *NR5A1* gene mutations in a large series of 46,XY DSD cases with phenotypes that ranged from complete gonadal dysgenesis to simple hypospadias. All cases showed no evidence of adrenal insufficiency. Five heterozygous *NR5A1* mutations were identified that were not observed in control individuals. Functional studies indicated altered biological activity for the mutant NR5A1 proteins. A *de novo* heterozygous mutation was identified in one individual with simple distal hypospadias. This extends the range of phenotypes associated with mutations in *NR5A1*.

## Materials and Methods

### Patient Recruitment

This retrospective study was performed on 77 patients, first seen from birth to 17 years of age, between 1981 and 2008 in a university pediatric hospital by one of us (R Brauner) for 46,XY DSD, including hypospadias.

The 77 patients were classified as complete or partial gonadal dysgenesis (uterus seen at genitography and/or surgery, n = 11), ambiguous external genitalia without uterus (n = 33) and hypospadias (n = 33). The patients classified as hypospadias had a normal penis length [greater than 30 mm, −2 SD, 21] and palpable inguinal or intrascrotal gonads bilaterally (n = 28) or unilaterally (n = 5). The meatus location was penoscrotal (n = 2), mid-shaft (n = 3) or glandular (n = 10).

Clinical examination included palpation of the labioscrotal and inguinal areas for the consistency of the gonads. Internal genitalia were evaluated by pelvic ultrasound examination and/or genitography. Associated abdominal ultrasound examination was carried out to assess kidney malformations. Several patients were given testosterone heptylate (4×100 mg/m^2^ given im every 15 days) in the neonatal period before the sex assignement and/or before the genitoplasty. For each patient, cytogenetic analysis was performed on peripheral blood leukocytes. The chromosome complement was determined by examining 40 to 50 metaphases from each patient. The endocrine evaluation was conducted to assess congenital adrenal hyperplasia and failure, testosterone biosynthesis by normal adrenocorticotropin hormone, cortisol, 17OH-progesterone, 11-deoxycortisol, dehydroepiandrosterone, and Δ4 androstenedione plasma concentrations. Leydig cell function was evaluated by measuring the basal plasma testosterone concentration and after stimulation with human chorionic gonadotropin (hCG, 3 or 7×1,500 IU) with samples taken the day after the last injection. In general, the hCG test was not performed in the patients with basal plasma testosterone concentration greater than 1 µg/L or with basal plasma luteinising hormone (LH) concentration greater than 5 IU/L. 5 α-réductase deficiency was investigated by measuring the testosterone/dihydrotestosterone ratio in basal situation or after stimulation with hCG. Aliquots of plasma were frozen at −20°C in the more recent patients to measure AMH (n = 58) and inhibin B (n = 45) and compared to the normal range for age [22], [23]–[26]. Plasma AMH was measured using the AMH/Mullerian-inhibiting substance ELISA kit (Immunotech-Beckman, Marseille, France). Plasma inhibin B levels were measured by ELISA using Oxford Bio-Innovation reagents (Diagnostic Systems Laboratories-France, Cergy-Pontoise, France). According to the manufacturer, sensitivity of the assay was 15 pg/ml; at a mean concentration of 100 pg/ml, the intra- and interassay coefficients of variation were less 6% and 16% respectively. Serum testosterone levels were measured by RIA after extraction using Orion reagents (Cis biointernational, Gif-sur-Yvette, France). Plasma LH and follicle stimulating hormone (FSH) levels were measured with an IRMA assay (Immunotech- Beckman, Marseille, France) with a sensitivity of 0.2 UI/L. The intra- and interassay coefficients of variation were less 6.7% in both gonadotropin assay. Conventional histological examination of the gonads was performed after gonadectomy of patients reared as females or following a gonadal biopsy (n = 12).

### Ethics Statement

This study was approved by the institutional review board and the local ethics committee (Comité de Protection des Personnes, Ile de France, III). Written, informed consent was given by all the parents for the evaluation including chromosomal and molecular biology analyses, and surgery.

### DNA analysis and Functional studies

Genomic DNA was extracted from peripheral blood leukocytes by standard procedures. The *NR5A1* gene was amplified and sequenced as described elsewhere [20]. NR5A1 expression vectors containing the p.Arg39Pro and p.Arg313Cys variants were generated by site-directed mutagenesis (QuikChange, Stratagene) using wild-type (WT) human NR5A1 cDNA in a pCMX expression vector as a template. Mouse full-length GATA4 cDNA was excised by digesting GATA4-GST vector (a gift from J. D. Molkentin, Cincinnati Children's Hospital Medical Center, Cincinnati) with EcoRI restriction endonuclease (New England Biolabs). The cDNA was then cloned in EcoRI-digested pIRES-hrGFP II vector (Stratagene). As a control the p.Gly35Glu NR5A1 mutant protein in vector in the expression pCMX was used (a gift from Dr J. C. Achermann, University College London, U.K.) [18]. The entire coding sequence of all mutant plasmids was confirmed by direct sequencing prior to functional studies. Transient gene expression assays to assess NR5A1 function were performed in 96-well plates (TPP) using human embryonic kidney cells HEK 393-T, FUGENE 6 (Roche), and a Dual-Luciferase reporter assay system (Promega) with pRLSV40 Renilla luciferase (Promega) vector as a marker of transfection efficiency. pCMXWT or mutant NR5A1 expression vectors (2 ng/well) were co-transfected into HEK 293-T cells with reporters containing SF1 responsive minimal promoters (murine *Cyp11a1*, human *AMH*; 10 ng/well) [27], [28]. Cells were lysed 24 hours later and luciferase assays were performed (Dual Luciferase Reporter Assay system, Promega) using a FLUOstar Optima fluorescence microplate reader (BMG Labtech). All data were standardized for Renilla activity. Results are shown as the mean±SEM of three independent experiments, each performed in triplicate.

### Far Western Analysis

Proteins were *in vitro* translated using the Quick Coupled TNT in vitro transcription and translation system (Promega) according to the manufacturer's protocols. Equivalent amounts of NR5A1 wildtype, NR5A1pGly35Glu, NR5A1p.Arg39Pro or NR5A1p.Arg313Cys were fractionated on a 10% SDS-PAGE gel and transferred to nitrocellulose membrane. The immobilised proteins were treated for 10 min each at room temperature with 6, 3, 1.5, 0.75, 0.38, and 0.19 M guanidine HCl solution containing 20 mM HEPES (pH 7.5), 50 mM NaCl, 1 mM EDTA, 1 mM DTT and 10% glycerol (Buffer A). Filters were blocked for 2 hours at room temperature in buffer A +1% BSA and then incubated with rocking overnight at 4°C with *in vitro* translated bait protein (GATA4 2 µg/ml) in buffer A (1% BSA and 0.1% Tween-20). The filters were washed three times with hybridization buffer and once with PBS-0.1% Tween-20. Bound bait was detected with anti-bait antibodies, (Abcam), followed by secondary antibody conjugated with horseradish peroxidase and visualized by chemiluminiscence using ECL reagent (Amersham).

## Results

### Mutations associated with 46,XY DSD

**Figure 1:**
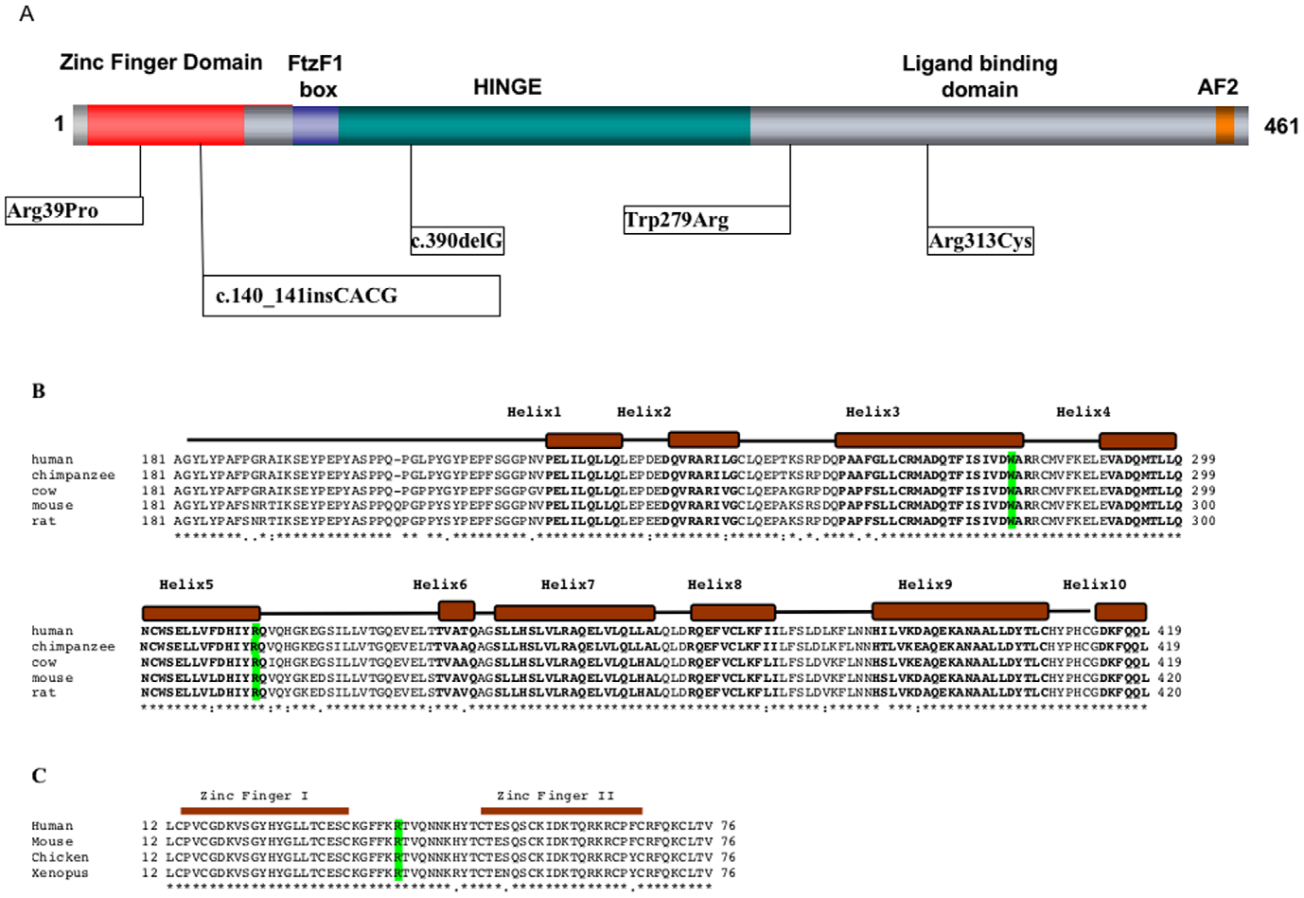
Mutations in *NR5A1* associated with 46,XY DSD. (A). Distribution of *NR5A1* mutations in relation to the functional domains of the protein. The DNA-binding domain (DBD) containing two zinc-finger motifs is indicated. The FtzF1 box stabilizes protein binding to DNA. The hinge region is important for stabilizing the ligand-binding domain and interacts with other proteins that control NR5A1 transcriptional activity. The AF2 domain recruits cofactors necessary for NR5A1 transactivating activity. (B) The sequence alignment of the distal portion of the hinge and the ligand-binding domain (LBD) of human NR5A1 protein as compared with those of other mammals. 1 to 10 of the predicted alpha helixes in the ligand-binding domain of NR5A1 are indicated as solid boxes, and corresponding amino acids in bold text. The position of the p.Trp279Arg and p.Arg313Cys are highlighted. The mutations fall either in the highly conserved Helix 3 (p.Trp279Arg) or in Helix 5 (p.Arg313Cys) of the ligand-binding domain. The mutation c.390delG was described previously [20]. (C) Evolutionary conservation of DNA binding domain of NR5A1. Sequence alignment of the evolutionary conserved DNA-binding domain of NR5A1 showing the position of the two zinc finger domains and the p.Arg39Pro mutation.

In the 11 cases of 46,XY with gonadal dysgenesis we did not detect a mutation in the *NR5A1* gene. In the 33 cases of 46,XY DSD with ambiguous external genitalia without uterus, we identified mutations in the *NR5A1* gene in four cases (12.1%; Table 1; Figure 1a). Case 1 carried a heterozygous T to A mutation at position that is predicted to result in a p.Trp279Arg amino acid change. This mutation is located in the evolutionary conserved helix 3 of the ligand-binding domain of the protein (Figure 1b). *In silico* analysis by Polyphen (http://genetics.bwh.harvard.edu/cgi-bin/ggi/ggi.cgi) indicated that this mutation is predicted to be highly damaging (PSIC score difference: 4.142). His apparently healthy mother carried this mutation. Case 2 carried a heterozygote G to C transition at position that is predicted to result in a p.Arg39Pro amino acid change within the N-terminal zinc finger region (Figure 1c). DNA samples from the parents of this case were unavailable for study and it is unknown if the mutation is inherited. In this case, at 6 years of age a laparatomy was performed that revealed bilateral testis of 20 mm diameter. Wolffian structures such as the epididymis were also present. The gonads were removed and the sex of rearing was female. Gonad histology showed the presence of cord structures with both Sertoli and Leydig cells present. Germ cells were not observed. Case 3 carries a heterozygous frameshift mutation c.390delG. This mutation is predicted to alter the protein sequence and create a premature termination codon in the mRNA at codon 295. The mother also carries the mutation and had premature menopause at 29 years. This familial case has been previously described (Family 4) [20]. In case 4, we identified a heterozygous four base pair insertion (CACG) at nucleotide position 140 within the zinc finger domain. This frameshift mutation is predicted to alter the protein sequence and create a premature termination codon in the mRNA at codon 87. The analyses of a DNA sample from the father showed a normal *NR5A1* sequence. DNA from the mother was unavailable for study. The sequence of the *SRY* gene was normal in all the XY individuals.

**Table 1:** Clinical and biochemical characteristics of five 46,XY DSD patients carrying a heterozygote mutations in *NR5A1*.

Patient	Declared Sex	Age at first evaluation	Location of Meatus	Genitalia	Endocrine data	*NR5A1* mutation
1	M	3 days	Penoscrotal	AmbiguousPhallus 20×10 mmTestis 17 mm,right in scrotum, left inguinalGenitography: vaginal rest 20 mm	hCG stimulation: testosterone 0.1 g–0.9 ng/ml after 3×1500 IU and 2.4 ng/mL after 6×1 500 IUFSH 5.2 IU/L. AMH 88 pmol/L. Inhibin B 109 pg/mL	p.Trp279Arg
2	F	6 years	Penoscrotal	Ambiguous, Prader III,Phallus 20 mm,Testis 20 mm in labiaSurgery: vaginal rest 15 mm	hCG stimulation: testosterone 0.05 g0.85 ng/mL	p.Arg39Pro
3	M	21 days	Penoscrotal	Ambiguous, Prader IIIPhallus 15×10 mmTestes in scrotum	hCG stimulation: testosterone 0.23 g0.9 ng/mL.Age 8.5 years, LH 0.3 IU/L, FSH 1.8 IU/L, AMH 78 pmol/L	c.390delG
4	M	1 day	Penoscrotal	Ambiguous, Prader IIIPhallus 17×7 mmGonads 18 mm in scrotum	Age 2 months; testosterone 2.9 ng/mL, LH 0.76 IU/LAge 4 months; testosterone <0.07 ng/mL, LH 0.41 IU/LAMH 398 pmol/L, Inhibin B 106 pg/mL	c.140_141insCACG
5	M	4 days	Glandular	HypospadiasPhallus 33×15 mmTestes 18 mm in scrotum.	Testosterone 1.2 ng/ml, LH 1.6 IU/L, FSH 0.5 IU/L, AMH 175 pmol/L, Inhibin B 99 pg/mL	p.Arg313Cys

Testosterone 0–4 months 0.1–3.5 ng/mL, 4 months–11 years <0.5 ng/mL; FSH 0–1 yr 1.5–3.5 IU/L; 1–10 yr 2.5–4.5 IU/L; LH 0–1 yr 1.5–4.5 IU/L; 1–10 yr 2–5 IU/L.

Inhibin B: 0–1 yr 94–383 pg/mL; 1–2 yr 71–204 pg/mL.

AMH<15 days 237.5+/−59.2, 15 days–1 year 464.8+/−92.8 pmol/L.

In the 33 cases of 46,XY DSD with hypospadias only, we identified a single *NR5A1* mutation (3%). This patient presented with distal hypospadias, normal phallus length, and a bifid scrotum containing testes that were normal in size and location. The mutation consisted of a C to T transition at nucleotide position 937 that is predicted to result in a p.Arg313Cys amino acid change. This mutation is located in the highly conserved helix 5 of the ligand-binding domain of the protein (Figure 1b). DNA was available from both parents and the mutation was found to be *de novo*. The sequence of the *SRY* gene was normal in all of these cases.

These mutations were not observed in the entire coding region of *NR5A1* in 270 fertile or 358 normospermic men, indicating that these mutations are associated with the phenotype [20], [29]. The *MAMLD1* gene on Xp28 was sequenced in 55 of the cases with ambiguous genitalia or hypospadias [4]. No pathogenic mutations were identified.

### Plasma AMH and inhibin B concentrations

**Figure 2:**
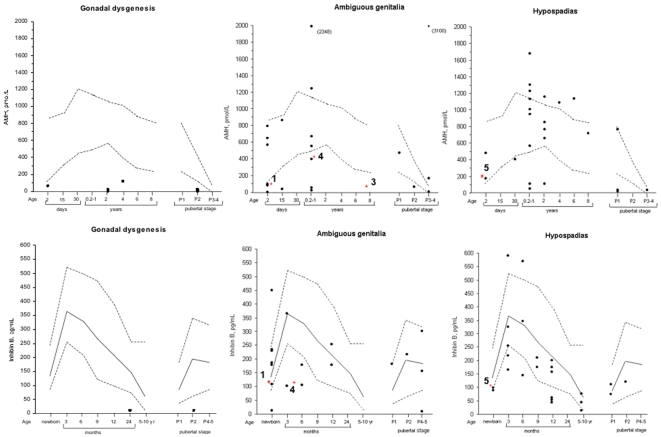
Plasma AMH and inhibin B concentrations in each patient group. In each AMH graph the broken lines correspond to the upper and lower limits of the normal range [22]. For inhibin B, the solid line corresponds to the median and the broken lines to the 5^th^ and 95^th^ percentiles [23], [24]. The red asterisk indicates individuals carrying an *NR5A1* heterozygote mutation and the numbers indicate the patients described in table 1.

Individuals with gonadal dysgenesis showed low AMH and low inhibin B levels consistent with the absence or reduced numbers of Sertoli cells and germ cells that is characteristic of the dysgenetic gonad (Fig. 2). Individuals without *NR5A1* mutations and either ambiguous external genitalia or hypospadias showed a much more variable aspect with plasma AMH and inhibin B levels below, within or above the normal range for age. Interestingly all individuals carrying heterozygote *NR5A1* mutations had plasma AMH and inhibin B levels that were under or at the lower limit of normal range irrespective of the phenotype (see also Table 1).

### Mutant NR5A1 proteins display reduced transactivation activity

**Figure 3:**
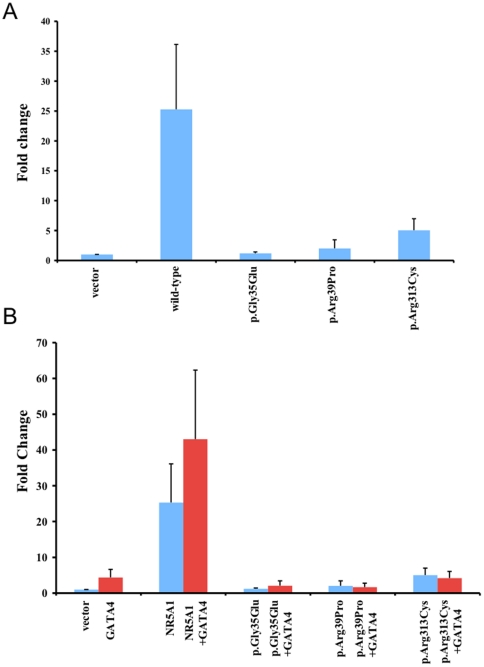
Assays of NR5A1 transactivation activity. The transcriptional activity of wild-type (WT) NR5A1 and mutant p.Gly35Glu (control) [18], p.Arg39Pro and p.Arg313Cys were studied using the human *AMH* promoter in HEK293-T cells. The human AMH reporter construct was transfected into HEK293-T cells with either the wild type or mutant NR5A1 expression vectors. Mutants p.Arg39Pro and p.Arg313Cys exhibited a dramatic reduction in transactivation activity (blue bars). The human AMH reporter construct was transfected into HEK293-T cells with either the wild type or mutant NR5A1 expression vector in the absence (blue) or presence (red) of the GATA4 expression vector. Data represent the mean±SEM of three independent experiments, each performed in triplicate. Results are expressed as fold activity of the empty vector activity.

The c.390delG mutation that results in a premature termination codon at amino acid position 295, predicted to be recognized by the nonsense-mediated decay surveillance complexes and degraded. We have previously demonstrated that even if a truncated protein were produced, it would have severely impaired transcriptional activity [20]. The heterozygous four base pair insertion (CACG) at nucleotide position 140 also results in the generation of a premature stop codon at codon position 87 and it is also predicted to undergo nonsense-mediated decay. We observed a quantitative reduction in the transactivation of both *CYP11A1* (not shown) and *AMH* promoters when assaying the effect of the NR5A1 mutants p.Arg39Pro and p.Arg313Cys on protein function, using human embryonic kidney HEK 293-T cells (Fig. 3). Similar to the previously published p.Gly35Glu protein [18], the p.Arg39Pro and p.Arg313Cys mutant proteins showed severe loss of activation. We obtained similar results in transient gene-expression assays using the mouse embryonic stem cell line E14 (data not shown).

### Mutant NR5A1 protein p.Arg39Pro and p.Arg313Cys physically interact with GATA4

**Figure 4:**
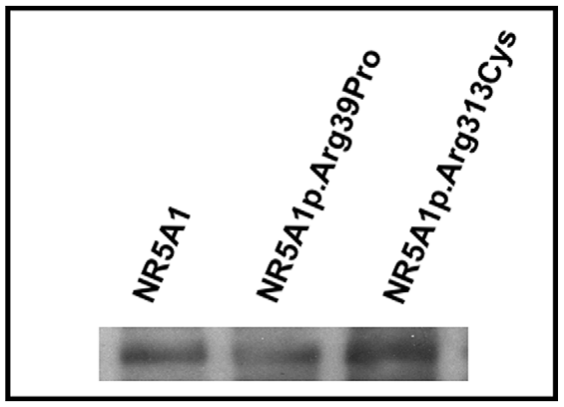
Far Western blot analysis of the interaction between *in-vitro* translated wild type GATA4 and wild-type NR5A1, NR5A1p.Arg39Pro and p.Arg313Cys. Blots containing wild-type NR5A1, NR5A1p.Arg39Pro and NR5A1p.Arg313Cys proteins were incubated with GATA4 protein and probed by anti-GATA4 antibody. Both the wild-type and mutant NR5A1 proteins can bind to GATA4 protein.

To further understand the mechanism whereby these mutations are associated with 46,XY DSD, we investigated whether the NR5A1 p.Arg39Pro and p.Arg313Cys mutant proteins physically interact with a known interacting protein GATA4. GATA4 functionally interacts with NR5A1 in primary Sertoli cell cultures and it has been shown that mutations in *NR5A1* may cause 46,XY DSD through a lack of appropriate interaction with *in vitro* translated GATA4 [30], [31]. Wildtype and mutant NR5A1 proteins were fractionated on a 10% SDS-PAGE gel and transferred to nitrocellulose membrane. The immobilised protein was incubated with *in vitro* translated bait protein (GATA4) and bound bait was detected with anti-bait antibodies, followed by secondary antibody conjugated with horseradish peroxidase and visualized by chemiluminiscence. In multiple experiments, the Far-Western analyses revealed that both mutant NR5A1 proteins retained their ability to interact with GATA4 (Figure 4).

### Mutant NR5A1 proteins p.Arg39Pro and p.Arg313Cys do not synergize with GATA4 to stimulate reporter gene activity

It is established that GATA4 and NR5A1 transcriptionally cooperate to synergistically activate the AMH promoter [30]. Although the mutant p.Arg39Pro and p.Arg313Cys proteins retained their ability to physically interact with the GATA4 protein (Fig. 4), they failed to synergize with GATA4 to stimulate *AMH* reporter gene activity in either HEK 293T cells (Fig. 3) or using the murine ES cell line E14 (data available upon request).

## Discussion

We have identified four novel missense and frameshift mutations in *NR5A1* associated with 46,XY DSD. All of these mutations were in heterozygous state and were associated with either ambiguous external genitalia or distal hypospadias in one case. Although mutations involving *NR5A1* have been reported associated with 46,XY gonadal dysgenesis in the absence of adrenal anomalies [14], [32] our data suggest that these mutations may be relatively uncommon in this group.

In contrast to the more severe phenotype of gonadal dysgenesis, we identified 5 *NR5A1* mutations in a heterozygous state in a series of 66 patients with various degrees of under androgenization. One of these cases was raised as a female whereas the other cases were raised as male and each presented with a range of anomalies of genital development (Table 1). We previously reported one of these mutations, which was familial and was also associated with ovarian insufficiency (c.390delG) [19]. In the other cases, the p.Arg313Cys is *de novo* and the p.Trp279Arg mutation was carried by the mother. The age of menopause of the mother is unknown and the ovarian function was not evaluated. The mode of transmission of remaining two mutations is unknown as other family members were not available for study.

*In vitro* and *in vivo* experiments have previously revealed that functional cooperation between NR5A1 and a protein partner GATA4 contribute to the proper spatiotemporal expression of the *AMH* gene during the development of mammalian genitalia [30], [31]. Our results indicate that the p.Arg39Pro and p.Arg313Cys NR5A1 proteins associated with ambiguous genitalia and distal hypospadias respectively showed a marked impairment of the function of the protein. Both the mutant proteins could physically interact with the GATA4 protein, but failed to synergise with GATA4 to stimulate *AMH* reporter gene activity. It is also possible that the phenotype could be due to a lack of synergy with SOX9 in the control of *AMH* expression [13]. Previously Lin et al., reported a boy (patient 4) with severe penoscrotal hypospadias and chordee, a small phallus and bilateral undescended testis who carried a heterozygous *de novo* L437Q *NR5A1* mutation [28]. Khöler et al., screened the *NR5A1* gene for mutations in a large series of 60 patients presenting with hypospadias. Of these 24 had mild penile hypospadias and 36 individuals had penoscrotal hypospadias with descended or undescended testes [19]. Three heterozygous mutations (8%) were identified (two nonsense mutations and a splice site mutation) in men with the more severe phenotype of penoscrotal hypospadias, variable degrees of androgenization of the phallus and undescended testes. Here, we have further extended the range of phenotypes associated with *NR5A1* mutations to include the milder phenotype of distal hypospadias. We did not identify mutations in the *MAMLD1* gene in a screen of 55 of the ambiguous genitalia/hypospadias cases. This is consistent with previous reports indicating that mutations involving *MAMLD1* are rare in hypospadias [33].

Where available, serum AMH and inhibin B levels were measured in each category of patient and compared with the normal range for age. Individuals with hypospadias or ambiguous external genitalia showed a wide variability in plasma concentrations for age. Although the number of 46,XY individuals carrying *NR5A1* mutations in this study is limited, it is interesting to note that *each* mutation is associated with plasma AMH or inhibin B levels that are less than, or at the limits of the normal range for age. These data suggest that the *NR5A1* mutations are associated with a primary testicular defect.

To date none of these patients have shown evidence of adrenal insufficiency but in case 2 the gonadal histology revealed a structurally normal testis with Leydig and Sertoli cells, although there was a complete absence of germ cells. This is consistent with our recent findings that mutations in *NR5A1* can lead to severe spermatogenic failure in 4% of otherwise unexplained cases of male infertility [29]. Our data show that *NR5A1* mutations can be associated with a wide spectrum of phenotypes including distal hypospadias and they highlight the need for a long-term follow up of this group of patients to see if signs of adrenal insufficiency or problems of fertility develop in later life.
